# 3D FRN-ResNet: An Automated Major Depressive Disorder Structural Magnetic Resonance Imaging Data Identification Framework

**DOI:** 10.3389/fnagi.2022.912283

**Published:** 2022-05-13

**Authors:** Jialin Hong, Yueqi Huang, Jianming Ye, Jianqing Wang, Xiaomei Xu, Yan Wu, Yi Li, Jialu Zhao, Ruipeng Li, Junlong Kang, Xiaobo Lai

**Affiliations:** ^1^School of Medical Technology and Information Engineering, Zhejiang Chinese Medical University, Hangzhou, China; ^2^Department of Psychiatry, Hangzhou Seventh People’s Hospital, Hangzhou, China; ^3^First Affiliated Hospital, Gannan Medical University, Ganzhou, China; ^4^Hangzhou Third People’s Hospital, Hangzhou, China; ^5^Zhongshan Hospital, Xiamen University, Xiamen, China; ^6^Department of Nephrology Surgery, Hangzhou Hospital of Traditional Chinese Medicine, Hangzhou, China

**Keywords:** major depressive disorder, deep learning, feature graph reconstruction network, structural magnetic resonance imaging, automated identification

## Abstract

Major Depressive Disorder (MDD) is the most prevalent psychiatric disorder, seriously affecting people’s quality of life. Manually identifying MDD from structural magnetic resonance imaging (sMRI) images is laborious and time-consuming due to the lack of clear physiological indicators. With the development of deep learning, many automated identification methods have been developed, but most of them stay in 2D images, resulting in poor performance. In addition, the heterogeneity of MDD also results in slightly different changes reflected in patients’ brain imaging, which constitutes a barrier to the study of MDD identification based on brain sMRI images. We propose an automated MDD identification framework in sMRI data (3D FRN-ResNet) to comprehensively address these challenges, which uses 3D-ResNet to extract features and reconstruct them based on feature maps. Notably, the 3D FRN-ResNet fully exploits the interlayer structure information in 3D sMRI data and preserves most of the spatial details as well as the location information when converting the extracted features into vectors. Furthermore, our model solves the feature map reconstruction problem in closed form to produce a straightforward and efficient classifier and dramatically improves model performance. We evaluate our framework on a private brain sMRI dataset of MDD patients. Experimental results show that the proposed model exhibits promising performance and outperforms the typical other methods, achieving the accuracy, recall, precision, and *F*1 values of 0.86776, 0.84237, 0.85333, and 0.84781, respectively.

## Introduction

Major Depressive Disorder (MDD), one of the most common diseases associated with suicidal behavior, has become increasingly prevalent in recent years and is expected to be the largest contributor to the world’s disease burden by 2030 (GBD 2017 Disease and Injury Incidence and Prevalence Collaborators, 2018). People with MDD are at higher risk for obesity, cardiovascular disease, stroke, diabetes, cognitive impairment, cancer, and Alzheimer’s disease. Approximately 8% of men and 15% of women suffer from depressive disorders during their lifetime, and nearly 15% of them choose to commit suicide (Gold et al., 2015). Therefore, it is crucial to diagnose MDD early and provide timely treatment.

Currently, the clinical diagnosis of MDD is mainly based on the relevant criteria in the Diagnostic and Statistical Manual of Mental Disorders (DSM), combined with the patient’s interview and the subjective judgment of the clinician (Sakai and Yamada, 2019). The rapid development of medical imaging technology has provided more possibilities for pathological and identification studies of psychiatric disorders. Common medical imaging available includes Computerized Tomography (CT), Positron Emission Tomography (PET), Magnetic Resonance Imaging (MRI). Compared with other types of medical images, brain structural MRI (sMRI) images can describe changes in brain tissue volume or structure and reflect changes in neural activity in the brain. Therefore, sMRI is widely used to detect and treat psychiatric disorders. On the other hand, Segall et al. (2009) have found that sMRI of the brain can generate reliable and accurate brain volume estimates, making it practical to study the classification of depression based on brain sMRI images. However, due to the lack of clear physiological indicators, images of MDD patients cannot visually present abnormalities or lesions. Therefore, automated MDD identification is urgently needed in clinical practice.

Under the deep learning method, it is not easy to obtain many training samples, and the heterogeneity of MDD is substantial. Furthermore, most current deep learning networks rarely involve 3D data. How to apply deep learning framework to the identification task of MDD sMRI data has become a research hotspot and challenge. So far, many outstanding studies have been presented, such as Seal et al. (2021) proposed a deep learning-based convolutional neural network named DeprNet to classify Electroencephalogram (EEG) data from MDD patients and normal subjects. Baek and Chung (2020) proposed a contextual Deep Neural Network (DNN) model using multiple regression to efficiently detect depression risk in MDD patients. However, the methods above use only a 2D deep convolutional neural network, which cannot obtain the image’s shallow and deep semantic features. It also easily leads to overfitting, which seriously affects the accuracy and robustness of the system and requires a considerable computational cost.

**FIGURE 1:**
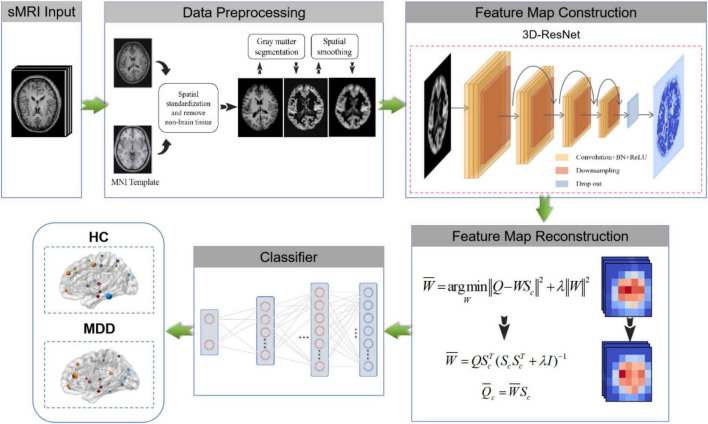
The overall diagram of our proposed 3D FRN-ResNet framework.

Previous methods rarely use sMRI data to identify MDD automatically and lack of MDD sMRI dataset, motivating us to start this study. Moreover, the primary purpose of this paper is to improve the automated identification accuracy of MDD effectively to help clinicians make a medical diagnosis. Therefore, we propose and develop an automated MDD sMRI data identification framework (3D FRN-ResNet), which introduces the Feature Map Reconstruction Network (FRN) based on the ResNet model. Its network structure is shown in Figure 1. Compared with other methods, our novel framework can preserve the granular information and details of the feature maps without overfitting the model. The contributions of our study are: (1) A feature map reconstruction network is proposed. (2) Building a 3D residual connectivity network to learn more deep features of sMRI images. (3) Preserving more texture details in sMRI images of MDD patients. (4) To get better identification results.

The remainder of this paper is organized as follows. After reviewing the state-of-the-art in the field of traditional machine learning-based methods, deep learning-based methods, and mental illness detection methods in Section “Related Works.” Then, we explain our approach for solving the problem of MDD identification with sMRI data in Section “Materials and Methods.” Then, we describe MDD sMRI dataset and the evaluation metrics, also the experimental details in Section “Experiments.” Finally, the results and the discussions are described in Sections “Results and Discussion,” followed by the conclusion in Section “Conclusion.”

## Related Works

### Traditional Machine Learning

In recent years, machine learning techniques have been widely used to mine medical images as computer-aided diagnostic methods. Multivariate pattern analysis (MVPA), a data-driven machine learning method, has been used in diagnostic classification studies of mental disorders at the individual level (Bachmann et al., 2017). Researchers have classified feature selection algorithms into Filter-style feature selection algorithms and Wrapper-style feature selection algorithms based on the different feature evaluation strategies (Lazli et al., 2019). In the Filter feature selection model, Mwangi et al. (2012) used the *T*-test algorithm to implement feature selection and classification on a multicenter MDD dataset. Moreover, in the Wrapper model, Guyon et al. (2002) proposed a support vector machine-based recursive feature elimination (RFE-SVM) algorithm for gene sequence feature selection. This algorithm has been widely used in machine learning tasks for medical image analysis, such as Hidalgo-Muñoz et al. (2014) used the RFE-SVM algorithm to classify structural image features of Alzheimer’s disease, which outperformed the *T*-test feature selection algorithm.

However, the Filter model usually has low computational intensity but poor classification accuracy; the Wrapper model has high classification accuracy but runs slowly, which is challenging to apply to datasets with many features. Therefore, researchers combined the advantages of both and proposed a combined Filter and Wrapper feature selection method to improve the classification accuracy while reducing the computational time overhead. Among them, Ding and Fu (2018) used the feature selection method combining the Filter model and Wrapper model to conduct experiments on several different types of datasets. The experimental results showed that the hybrid algorithm has high computational efficiency and classification accuracy (Ding and Fu, 2018). However, the drawback of the above methods is that they usually require manual feature design and redundant feature removal to extract useful distinguishable features.

### Deep Learning

Deep learning techniques have led to remarkable progress in machine learning methods and promising results in medical image classification applications. Chen et al. (2021) proposed a cyclic Convolutional Neural Network (CNN) framework that can take full advantage of multi-scale and multi-location contexts in a single-layer convolution (LeCun et al., 1989). Cyclic CNNs can be easily plugged into many existing CNN pipelines, such as the ResNet family (He et al., 2016), resulting in highly low-cost performance gains (Chen et al., 2021). Liang and Wang (2022) proposed a novel model which uses involution and convolution (I-CNet) to improve the accuracy of image classification tasks by extracting feature representations on the channel and spatial domains. Wang et al. (2021) proposed a semi-supervised generative adversarial network (CCS-GAN) for image classification. It employs a new cluster consistency loss to constrain its classifier to maintain local discriminative consistency in each unlabeled image cluster. At the same time, an enhanced feature matching approach is used to encourage its generator to generate adversarial images from low-density regions of the true distribution, thus enhancing the discriminative ability of the classifier during adversarial training. The model achieves a competitive performance in semi-supervised image classification tasks (Wang et al., 2021). For fine-grained image classification, it has been a challenge to quickly and efficiently focus on the subtle discriminative details that make subclasses different from each other. Zhang et al. (2021) proposed a new multi-scale erasure and confusion method (MSEC) to address the challenge of fine-grained image classification.

Furthermore, Dai et al. (2021) proposed a model named TransMed for multimodal medical image classification in terms of medical image. TransMed combines the advantages of CNN and transformer to efficiently extract low-level features of images and establish long-range dependencies between modalities. The method has great potential to be applied to many medical image analysis tasks. Karthikeyan et al. (2020) used three pre-trained models-VGG16 (Simonyan and Zisserman, 2014), VGG19 (Simonyan and Zisserman, 2014), RESNET101 (He et al., 2016), on a dataset of X-ray images from patients with common bacterial pneumonia, COVID-19 patients, and healthy individuals to investigate migration learning methods. The proposed method obtained the best results (Karthikeyan et al., 2020). Talaat et al. (2020) proposed an improved hybrid image classification method that uses CNN for feature extraction and a swarm-based feature selection algorithm to select relevant features.

### Mental Illness Detection

There are numerous mental illness detection algorithms, most of which are based on improvements to the basic deep learning framework. Payan and Montana (2015) used sparse autoencoder and 3D convolutional neural networks based on the Alzheimer’s Disease Neuroimaging Initiative (ADNI) datasets to build algorithms that could predict patients’ disease status, outperforming the latest research findings at the time. Similarly, Farooq et al. (2017) applied deep convolutional neural networks such as Goolenet and ResNet on the ADNI dataset to learn discriminative features, achieving the purpose of classifying Alzheimer’s disease (AD), mild cognitive impairment (MCI), advanced mild cognitive impairment (LMCI), and healthy individuals. Moreover, the prediction accuracy of the proposed technique was significantly improved compared (Farooq et al., 2017). Li and Liu (2018) applied the deep dense network (DenseNet) to the ADNI dataset. The original sMRI images did not need to be standardized preprocessing and directly extracted and classified features. The experimental results proved the effectiveness of the proposed method. Yang et al. (2022) proposed a spatial similarity-based perceptual learning and fusion deep polynomial network model to learn further robust information to detect obsessive-compulsive disorder (OCD); the model achieved promising performance in the rs-fMRI dataset of OCD patients. Ulloa et al. (2015) proposed a classification architecture using synthetic sMRI scans to scale up the sample size efficiently. A simulator that can capture statistical properties from observed data using independent component analysis (ICA) and random variable sampling methods was also designed to generate synthetic samples. Afterward, the DNN was specially trained on continuously generated synthetic data, and it significantly improved the generalization ability in classifying Schizophrenia patients and healthy individuals (Ulloa et al., 2015). Eslami et al. (2019) devised a data augmentation strategy to generate the synthetic dataset required to train the ASD-DiagNet model. The model consists of an auto-encoder and single-layer perceptron to improve the quality of extracted features and improve the detection efficiency of autism spectrum disorder.

### Our Work

Although various deep learning frameworks have been proposed and significant progress has been made in the classification of brain tumor images. There are still challenges, such as insufficient sample size for training (Wertheimer et al., 2021), overfitting or underfitting due to the increased dimensionality of images (from 2D to 3D), and excessive consumption of computational resources (Pathak et al., 2019). In addition, the use of deep learning feature representation has weakened the interpretability of the features and is not conducive to the pathological analysis and understanding of the learned features (Zadeh Shirazi et al., 2020). These challenges limit the application of deep learning in medical images, so more innovative deep learning models are needed to achieve better results in medical images.

We propose a 3D FRN-ResNet framework for MDD sMRI images identification, which uses 3D-ResNet as the base framework. The conventional ResNet network incorporates pooling operations to extract global features, discarding a large amount of local detail information and thus reducing the resolution of the data. Specifically, during sMRI image processing of the brain, changes in neural activity in abnormally active (or inactive) brain regions are difficult to capture, but these small changes may be necessary for MDD. To solve this problem, we introduce the FRN method so that the granularity information and details of the feature map can be retained without overfitting the model. Its network structure is shown in Figure 1. It achieves this by framing class membership as a problem in reconstructing the feature map. Given a set of images belonging to a single class, we generate the associated feature maps and collect the component feature vectors across locations and images into a single pool of support features. For each query image, we attempt to reconstruct each location in the feature map as a weighted sum of the support features with a negative mean squared reconstruction error as the class score. Images from the same class should be easier to reconstruct because their feature maps contain similar embeddings, while images from different classes are more complex and produce larger reconstruction errors. By evaluating the reconstruction of the complete feature map, FRN preserves the spatial details of the appearance. Additionally, by allowing this reconstruction to use feature vectors from any location in the support image, FRN explicitly discards the annoying location information. An auxiliary loss function is also introduced, which encourages orthogonality between features of different classes to focus on feature differences.

We evaluate the performance of the proposed model on a constructed sMRI dataset of MDD patients and compare it with other methods. The results show that our model has good performance in automated MDD sMRI data identification. (1) A novel identification network structure based on feature map reconstruction is proposed in this paper. (2) Feature extraction followed by feature map reconstruction of sMRI images retains more fine spatial details and dramatically improves the identification performance. (3) Classification-assisted loss functions are developed to distinguish between different features classes.

## Materials and Methods

Our goal is to identify MDD using sMRI images automatically. In order to obtain good identification performance, a robust network structure is usually required. Therefore, we propose the 3D FRN-ResNet model for automated MDD sMRI data identification, consisting of a feature extraction network and a feature map reconstruction network. The network structure of 3D FRN-ResNet is shown in Figure 1. This section describes the preprocessing process, the network structure of 3D FRN-ResNet, and the loss function used in detail.

### Data Preprocessing

The sMRI data preprocessing work is implemented using the MATLAB-based SPM12 toolkit (Ashburner et al., 2021). The main contents of preprocessing include AC-PC calibration, non-brain tissue removal, gray matter segmentation, spatial standardization, and spatial smoothing. The size of sMRI data for each subject after processing is 121 × 145 × 122 voxels.

#### Anterior Commissure-Posterior Commissure Calibration

The calibration procedure focuses on the anterior commissure (AC) and posterior commissure (PC) calibration. We use MATLAB software to perform AC-PC calibration, resampling the images in the standard 256 × 256 × 256 mode, and then the N3 algorithm is used to correct for non-uniform tissue intensity. We also perform skull stripping and cerebellar resection after correcting the images by AC-PC correction.

#### Non-brain Tissue Removal

**FIGURE 2:**
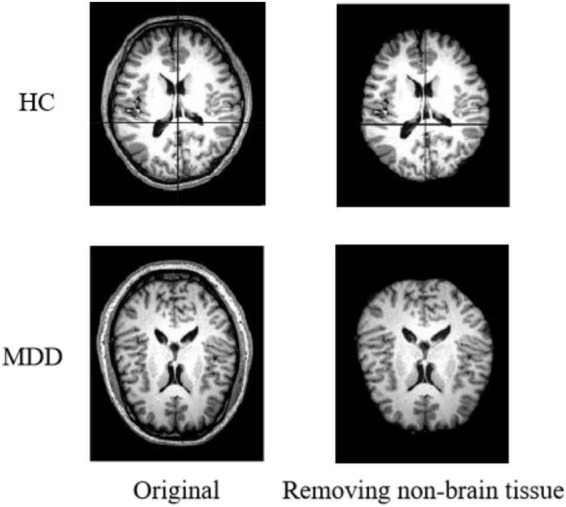
Results of removing non-brain tissue.

The original images of sMRI contain some non-brain structures, such as skulls. In order to avoid increasing the computational workload and to avoid subsequent image preprocessing, which may affect the experimental results. Non-brain structures such as skulls need be removed from the images during the image preprocessing operation. Figure 2 shows the comparison of a sample before and after removing non-brain tissue.

#### Gray Matter Segmentation

**FIGURE 3:**
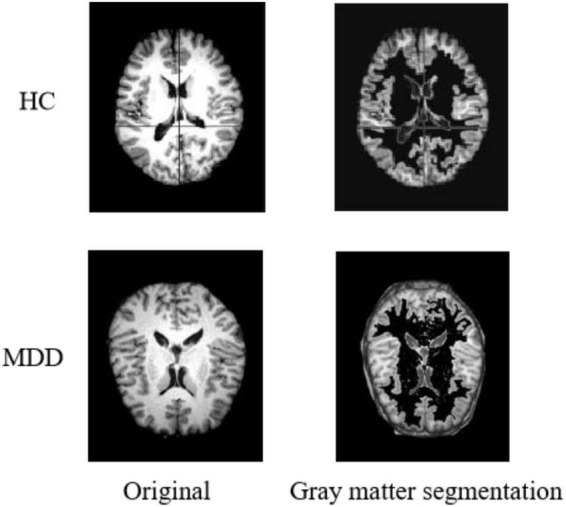
Results of gray matter segmentation.

During sMRI image processing, sometimes only the state of specific regions is focused on, which requires tissue extraction from the target area according to the brain’s anatomy. In the preprocessing process, we segment the sMRI into three different images by brain gray matter, white matter, and cerebrospinal fluid structures. Considering the critical influence of the gray matter region on the diagnosis of MDD (Arnone et al., 2013), only the gray matter part is used for the experiments in this paper. Figure 3 shows the result of gray matter segmentation.

#### Spatial Standardization

**FIGURE 4:**
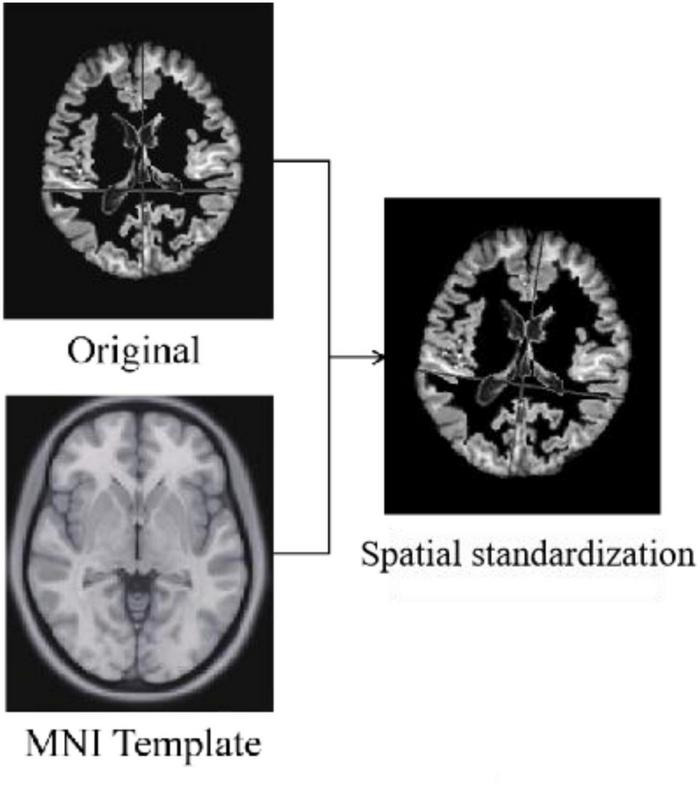
Results of spatial standardization.

Standardization is the alignment of the images from the previous preprocessing process to the standard brain template space Montreal Neurological Institute (MNI) to unify the coordinate space of all images. The algorithms used for standardization are non-rigid body alignment algorithms, including affine and non-linear transformations. Figure 4 shows the comparison of a sample before and after spatial standardization.

#### Spatial Smoothing

**FIGURE 5:**
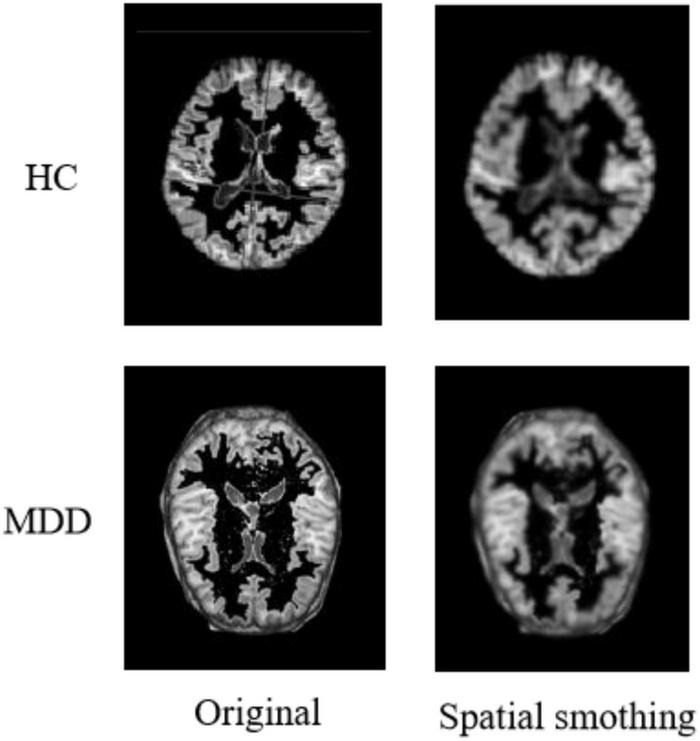
Results of spatial smoothing.

After completing the above series of processing, it is also necessary to perform a smoothing process on the image to suppress the noise of the functional image. Additionally, the signal-to-noise ratio needs to be improved to reduce anatomical or functional differences between images. Usually, the function used for the smoothing process is the Gaussian kernel function. In addition, based on experience and practical attempts, we use a 64 × 64 × 64 pixel cube to down-sample gray matter density images, and this processing saves computing time and memory consumption with no loss of classification accuracy. Figure 5 shows the comparison of a sample before and after spatial smoothing.

### 3D-ResNet Framework

Although ResNet has achieved excellent results on many 2D natural image datasets, it has little success in medical images. The reason is that the convolution kernels and pooling kernels in 2D networks are two-dimensional matrices. It can only move in the two directions of height *H* and width *W* of 2D flat images, so only 2D features can be extracted. In contrast, most medical image data such as sMRI are 3D stereo data. When using 2D network processing, only 3D images can be input in layers, or one of the dimensions can be used as the channel dimension. But neither of the two methods can make good use of the inter-layer structure information of the data.

**FIGURE 6:**
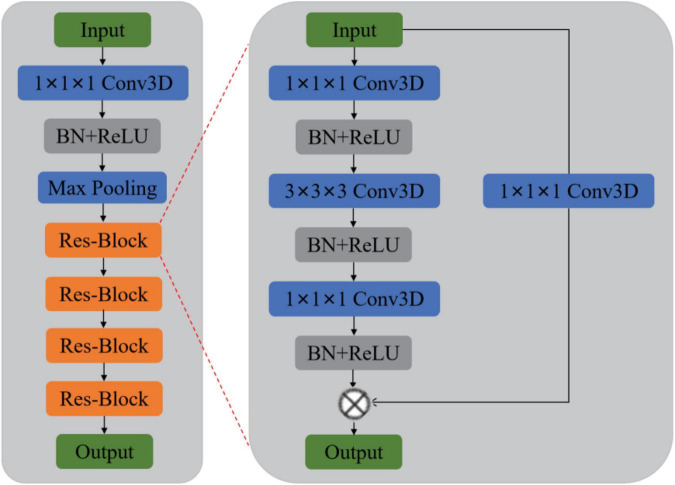
Proposed 3D-ResNet structure.


Based on this, this paper adds a depth dimension *D* to the filters such as convolution kernels and pooling kernels in the 2D network, and extends them into 3D matrix. In this way, the filters can be moved in all 3 directions (*H*, *W*, *D*) of the sMRI data, so that the spatial information of the data can be fully exploited. And the output of each filter is also a 3D data. The structure diagram of the 3D-ResNet is shown in Figure 6. Let the size of one of the 3D convolution kernels is *k* × *k* × *k* × *channel*, the number is *n*, the input data size is *h* × *w* × *d.* And since the sMRI data used in this paper is similar to a grayscale map, the channel dimension is 1. Therefore, the output size of this convolution kernel is as follow:

(1) (h-k+1)×(w-k+1)×(d-k+1)×n

By a similar method, the pooling layer and batch normalization layer in ResNet can be extended to construct a 3D residual connected network (3D-ResNet). The network can better extract representative features from 3D sMRI data and improve the accuracy of identification in MDD patients.

The 3D-ResNet network structure is shown in Figure 6. Due to the small size of the input region feature map, the convolution pooling operation is removed from the bottom layer of the network. And the input map is directly made to enter the residual network consisting of four stacked residual convolution modules.

**FIGURE 7:**
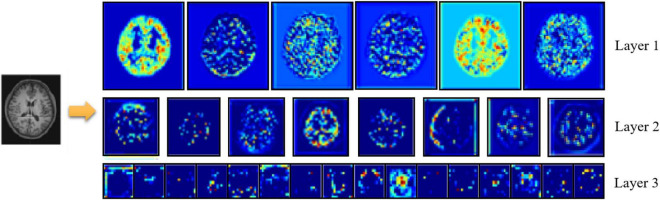
Visualization of extracted features.

Figure 7 shows an example of feature extraction from the 3D-ResNet middle layer. At the end of the extraction process, the network learns details such as contour boundaries, position, and orientation, enabling more learning of deeper features in the sMRI and preparing it for the next step.

### Feature Map Reconstruction Networks Framework

The feature extractor can produce a feature map. However, the distance metric function requires a vector representation of the whole graph. Therefore, a method needs to be found to convert the feature map into a vector representation. Ideally, this conversion would preserve the granularity of information and details of the feature map without overfitting the model. But existing methods, such as global average pooling, are very crude in discarding some spatial information or flattening a feature map into a long vector, which also loses location information. In order to convert the feature map into a vector representation while preserving the spatial details, Feature Map Reconstruction Networks (FRN) are proposed in this paper.

When there is a single input image *x*_*q*_, we wish to predict its label *y*_*q*_. Firstly, let *x*_*q*_ passes through feature extractor to generate a feature map *Q* ∈ ^R*r*×*d*^, where *r* represents the size of the space and *d* is the number of channels. For each class *c* ∈ *C*, we pool all features from the *k* input images into a feature matrix *S*_*c*_ ∈ *R^kr^*×*^d^*.

Then, we try to reconstruct *Q* as a weighted sum of rows in *S*_*c*_ by finding the matrix *W* ∈ *Rr*×*kr* so that *W*×*S*_*c*_≈*Q* can be obtained. Finding the optimal $\bar{W}$ is equivalent to solving the linear least squares problem:


(2) $$\bar{W}=\underset{W}{\arg\min}{||{\text{Q-WS}}_{c}||}^{2}+\lambda \,{||W||}^{2}$$

where || • || is the Frobenius norm, which λ is a weighted ridge regression penalty term used to ensure the treatability of the linear system when it is over- or under-constrained (*kr* ≠ *d*).

The ridge regression equation leads to the optimal solution *W* and *Q*_*c*_.


(3) $$\bar{W}=Q\,S_{c}^{T}\,{\left(S_{c}\,S_{c}^{T}+\lambda \,I\right)}^{-1}$$

(4) $${\bar{Q}}_{c}=\bar{W}\,S_{c}$$

For a given class *c*, the distance between *Q* and *Q*_*c*_ is defined as the Euclidean distance and then deflated by using $\frac{1}{r}$. A learnable temperature factor λ is also introduced. The final predicted probability is thus given by:


(5) $$\left\langle Q,{\bar{Q}}_{c}\right\rangle =\frac{1}{r}\,{||Q-{\bar{Q}}_{c}||}^{2}$$

(6) $$P\left(y_{q}=c|x_{q}\right)=\frac{e^{\left(-\gamma \,\left\langle Q,{\bar{Q}}_{c}\right\rangle \right)}}{\sum_{c^{\prime }\in C}e^{\left(-\gamma \,\left\langle Q,{\bar{Q}}_{c^{\prime }}\right\rangle \right)}}$$

In order to ensure the stability of the training, we decide to use $\frac{1}{k\,r}$ to improve λ. This has the additional benefit of making our model somewhat robust, in addition to the parameters that λ should be learned. The change λ has diverse effects: the large one λ avoids over-reliance on the weights of *W*, but it also reduces the effectiveness of the reconstruction. And it increases the reconstruction errors as well as limit the distinguishability. Therefore, we disentangle the degree of regularization ρ from the magnitude of *Q*_*c*_ by introducing a learned recalibration term. This leads to the following formula:


(7) $${\bar{Q}}_{c}=\rho \,\bar{W}\,S_{c}$$

λ andρare parameterized as *e*^α^ and *e*^β^ to ensure non-negativity and are initialized to zero. In summary, our final prediction is given by the following equation.


(8) $$\lambda =\frac{k\,r}{d}\,e^{\alpha }\;\rho =e^{\beta }\,$$

(9) $${\bar{Q}}_{c}=\rho \,\bar{W}\,S_{c}=\rho \,Q\,S_{c}^{T}\,{\left(S_{c}\,S_{c}^{T}+\lambda \,I\right)}^{-1}\,S_{c}$$

(10) $$P\left(y_{q}=c|x_{q}\right)=\frac{e^{\left(-\gamma \,\left\langle Q,{\bar{Q}}_{c}\right\rangle \right)}}{\sum_{c^{\prime }\in C}e^{\left(-\gamma \,\left\langle Q,{\bar{Q}}_{c^{\prime }}\right\rangle \right)}}$$

The method introduces only three learning parameters:α,β, andγ. The temperature γ appears in previous works (Chen et al., 2020).

**FIGURE 8:**
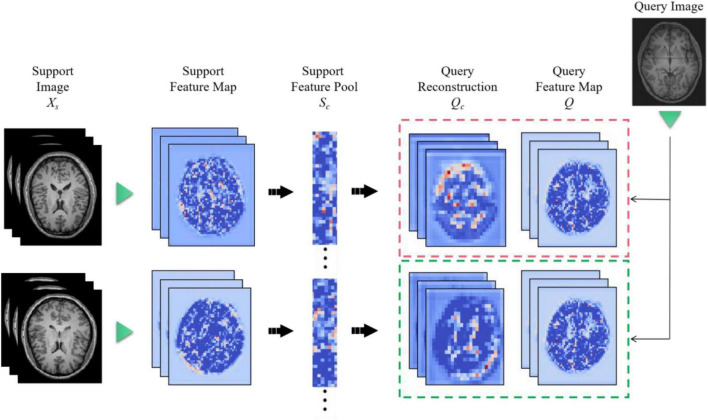
Feature map reconstruction networks network structure diagram.

Figure 8 is a diagram of the FRN network structure. Support image is converted to a feature map (left) and aggregated to a pool of class conditions (middle). A best-fit reconstruction of the query feature map is computed for each class, and the closest candidate generates the predicted class (right). Among them, *h* × *w* is the feature map resolution, *d* is the number of channels, and the green triangle represents the convolutional feature extractor.

### Loss Function

Medical image classification often faces the problem of minor differences in the appearance of pathological targets and non-targets. We also face this challenge for our MDD brain tumor classification task. For this purpose, our loss function consists of two components. The first is the cross-entropy loss function, which can be understood as a composition of two parts. The first part is the calculation of the mutual entropy with label 1, and the second part is the calculation of the mutual entropy with label 0. We sum the two to obtain the overall mutual entropy. The formula is as follows.


(11) $$L=-\frac{1}{N}\,\sum_{i=1}^{N}\left[y_{i}\,\log\left(p_{i}\right)+\left(1-y_{i}\right)\,\log\left(1-p_{i}\right)\right]$$

where *N* is the total number of samples, *y*_*i*_ is the category to which the *ith* sample belongs, and *p*_*i*_ is the predicted value of the *ith* sample.

In addition to the classification loss, we use an auxiliary loss that encourages support features from different classes to span the potential space.


(12) $$L_{a\,u\,x}=\sum_{i\in C}\sum_{j\in C,j\neq i}{||{\hat{S}}_{i}\,{\hat{S}}_{j}^{T}||}^{2}$$

Among then, $\hat{S}$ is line normalized and projects the features onto the unit sphere. This loss encourages orthogonality between features from different classes. Similar to Christian et al. (2020), we reduce this loss by a factor of 0.03. We use *L*_*aux*_ as the auxiliary loss in our subspace network implementation, which replaces the SimCLR fragment in the cross-transformer implementation (Carl et al., 2020).

## Experiments

### Dataset

**FIGURE 9:**
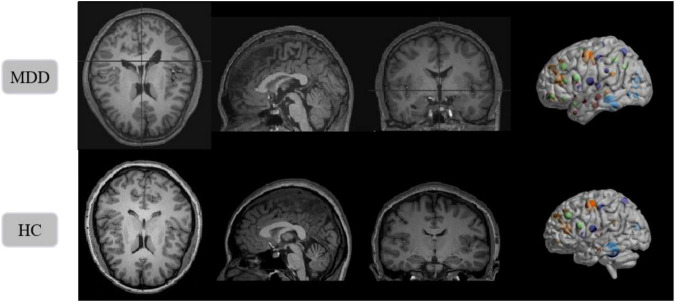
Structural magnetic resonance imaging slice images from the MDD and HC in SHH dataset. Left to right: axial view, sagittal view, coronal view, and 3D presentation.

The benchmarking clinical MDD sMRI images dataset is collected at the Seventh Hospital of Hangzhou (SHH) with Institutional Review Board (IRB) approval, and is used to train and test our model. Furthermore, the SHH dataset contains 68 subjects, including 34 MDD patients and 34 healthy controls (HC). All patients with MDD met the diagnostic criteria of the Diagnostic and Statistical Manual of Mental Disorders, Fourth Edition (DSM-IV) for MDD. And all healthy controls passed the non-patient version of the structured clinical interview of the DSM-IV. All sMRI images have an imaging field of view (FOV) = 240 mm × 256 mm, a voxel size of 1 mm × 1 mm × 1 mm, a layer thickness of 1 mm, and a scan layer count of 192. sMRI slice images from the MDD and HC in SHH dataset are shown in Figure 9.

### Evaluation Metrics

A total of 54 samples in SHH dataset are used in the training process, including 27 MDD patients and 27 healthy individuals. In addition, 14 samples are used for validation, including 7 MDD patients and 7 healthy individuals. We use four metrics to evaluate the model performance: Accuracy, Recall, Precision, and *F*1 score. Accuracy is calculated as:


(13) $$A\,c\,c\,u\,r\,a\,c\,y=\frac{T\,N+T\,P}{F\,P+T\,N+T\,P+F\,N}$$

where TN, TP, FP, and FN are the number of true negative, true positive, false positive, and false negative, respectively. Recall refers to the ability of a classifier to correctly detect positive samples, reflecting the proportion of patients with MDD that are correctly determined as a percentage of the total number of patients, defined as:


(14) $$R\,e\,c\,a\,l\,l=\frac{T\,P}{T\,P+F\,N}$$

Precision refers to the proportion of samples with a positive prediction that are correctly predicted, defined as:


(15) $$P\,r\,e\,c\,i\,s\,i\,o\,n=\frac{T\,P}{T\,P+F\,P}$$

Precision and Recall are contradictory metrics. In general, Recall tends to be low when Precision is high, while Recall tends to be high when Precision is low. When the classification confidence is high, Precision is high; when the classification confidence is low, Recall is high. To be able to consider these two metrics together, the weighted average F-measure of Precision and Recall is proposed, which reflects the overall metric, defined as:


(16) $$F\,1=\frac{2\times P\,r\,e\,c\,i\,s\,i\,o\,n\times R\,e\,c\,a\,l\,l}{P\,r\,e\,c\,i\,s\,i\,o\,n+R\,e\,c\,a\,l\,l}$$

In disease diagnosis studies, the higher the recall rate, the smaller the missed diagnosis rate. Therefore, the accuracy and recall of models are of most interest.

### Experimental Details

In deep learning training, the setting of hyperparameters is critical and determines the performance of our model. In the training of the 3D FRN-ResNet model, the initial learning rate is set to 0.01, the weight decay value is set to 0.001, the number of epochs is 100, and then the learning rate is changed to 0.1 times when the validation set loss value does not drop for 10 consecutive epochs. Considering the sample size limitation and using a fivefold cross-validation method to enhance the model’s generalization ability.

All experiments are performed on a CentOS server with NVIDIA TITAN Xp GPU, dual-core Intel(R) Xeon(R) Silver 4210 CPU @ 2.20 GHz processor, Python 3.6 programming language, and PyTorch 1.0 deep learning framework.

## Results

We use four metrics, Accuracy, Recall, Precision, and *F*1 value, to measure model performance. The average results of the metrics obtained on the training and validation sets are shown in Table 1. The experimental results show that the model has good robustness. We can observe that the Recall is at a high value, which indicates that the model is quite comprehensive in MDD patient identification. Furthermore, we can see that the Precision is also at a high value, demonstrating that the model has a good ability in MDD patient identification. In addition, the Recall of the training set is 0.84, and the *F*1 value of the training set is 0.85, which is very close. The same is true in the validation set, suggesting the ability to discriminate between healthy and MDD patients in our model is about the same.

**TABLE 1:** Test results on the training and validation sets.

	Accuracy	Recall	Precision	*F*1
Training	0.86	0.84	0.85	0.85
Validation	0.78	0.76	0.77	0.76

**FIGURE 10:**
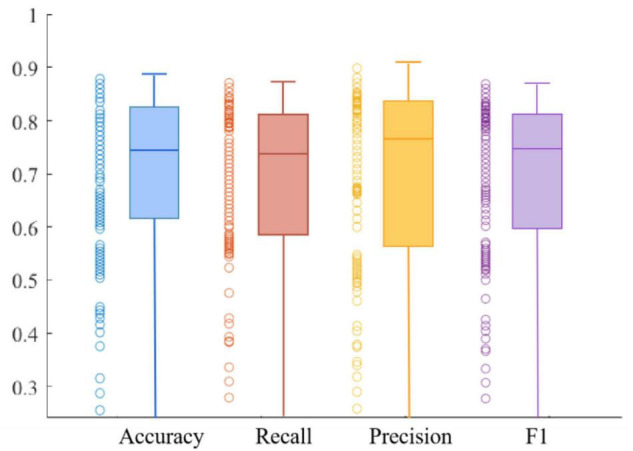
Combination of scatter plot and box plot of training set.

**FIGURE 11:**
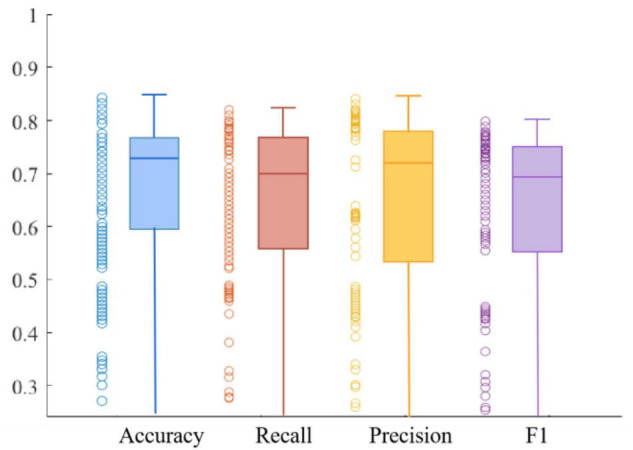
Combination of scatter plot and box plot of validation set.

Figures 10, 11 respectively show the composite plot of the scatter plot and box plot of the evaluation index results of the training set and the validation set. It can be seen from the box plots that the fluctuations of the results are tiny, and only a few outliers appear. In the box plot, the horizontal line in the middle of the box indicates the median of a dataset. It can also be observed in the scatter plot that the recall rate reaches a high range, and the recall rate represents the ability of the model to diagnose patients who suffer from MDD. The smaller the difference between the Recall and *F*1 values, the better the model’s performance in resolving class imbalance. It can be seen from the figure below that the recall fluctuation range is not large, indicating that the model has the similar ability to predict the MDD patients and healthy individuals. After validation, the overall performance of the model reached a high level.

In order to explore the influence of different network structures on the performance of the MDD identification algorithm, firstly we use five feature extraction networks with different structures for training based on the FRN structure in the classification layer. After that, the 3D-Resnet structure with the best effect is used as the feature extraction network, and the FRN structure is replaced with a general fully connected layer for classification. The experimental results show that compared with ordinary convolutional networks, ResNet and DenseNet structures can extract and retain richer detail information, and learn feature representations with strong discriminative power, thereby effectively improving the identification accuracy of the network.

From Table 2, we can see that the structural model combining 3D-ResNet and FRN has the highest classification accuracy, with the correct rate and recall rate achieving 85 and 84%, respectively. We can also observe that accuracy and recall have been significantly improved after the 3D operation of the network. For example, the identification accuracy of 3D-ResNet is 6% higher than that of 2D DenseNet, which shows that the 3D-ResNet proposed in this paper can mine effective information, providing more effective features than the general ResNet and the traditional 2D networks. Meanwhile, it can be seen from Table 2 that the FRN network can effectively improve the high heterogeneity problem in the sMRI images of MDD patients and thus is applicable in MDD sMRI images identification.

**TABLE 2:** Results comparison with different network structures.

Model	Backbone	Accuracy	Recall	Precision	*F*1
FRN(ours)	3D-ResNet	**0.85**	**0.84**	0.86	**0.84**
	ResNet	0.79	0.78	0.80	0.79
	3D-DenseNet	0.84	0.82	**0.87**	0.84
	DenseNet	0.78	0.78	0.79	0.78
	SimpleCNN	0.60	0.58	0.61	0.60
Full connected	3D-ResNet	0.82	0.80	0.82	0.81

*Bold values mean the best performance.*

**FIGURE 12:**
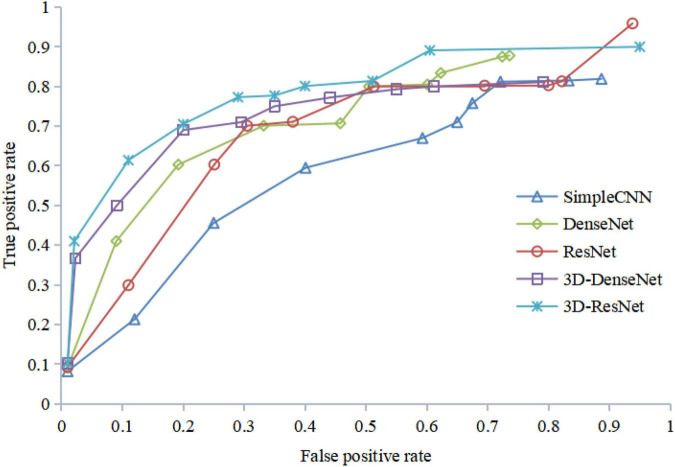
ROC curves with FRN-Net for different backbones of the training set.

Figure 12 shows the ROC curves of different algorithms using FRN-net on the SHH dataset. It can be seen that from the figure out algorithms outperforms others, which further confirms the effectiveness of our algorithm. The main reason is that we exploit both multi-scale layers and contextual spatial information to reduce the semantic gap to a large extent.

**FIGURE 13:**
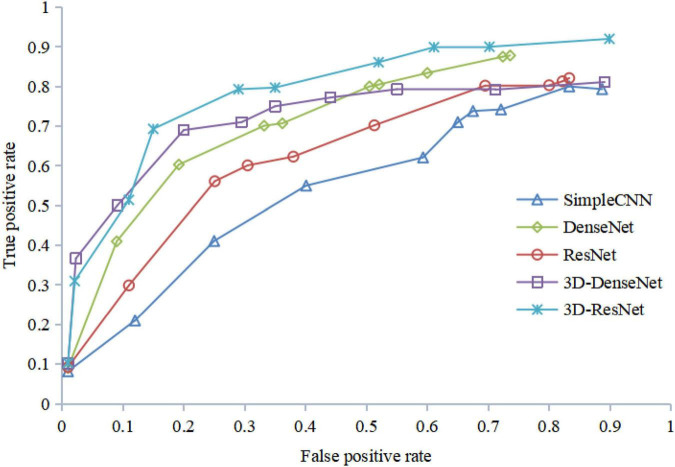
ROC curves with FRN-Net for different backbones of the validation set.

The results of the ROC curve in Figure 13 are consistent with those in Figure 12, indicating that our algorithm does improve image identification accuracy. On the one hand, our algorithm proposes a 3D residual connection network, which extends the idea of residual connections to three dimensions. It makes full use of the spatial and contextual information of the image, and preserves the spatial details when converting the extracted features into vectors and location information. Thus, higher average accuracy than other methods is achieved, which also demonstrate the effectiveness of the 3D residual connection network and classification based on feature map reconstruction. On the other hand, since we decompose the image into multiple-scale layers, sufficient scale information is used when generating multi-scale visual histograms. Therefore, our method has the best classification specificity and sensitivity.

To further illustrate that the feature map reconstruction method proposed in this paper is informative for correct classification, we obtain experimental results for each query image. In Table 3, all networks are trained with 3D-ResNet as the backbone. The results in their tables validate the effectiveness of the classification method based on the feature map reconstruction proposed in this paper.

**TABLE 3:** Results comparison with different classifiers.

	Model	Accuracy	Recall	Precision	*F*1
Train	ProtoNet	0.82	0.81	0.83	0.82
	DSN	0.81	0.79	0.82	0.81
	CTX	0.80	0.79	0.81	0.80
	**FRN(ours)**	**0.86**	**0.83**	**0.84**	**0.83**
Validation	ProtoNet	0.76	0.75	0.77	0.76
	DSN	0.74	0.72	0.75	0.74
	CTX	0.75	0.74	0.76	0.75
	**FRN(ours)**	**0.80**	**0.78**	**0.76**	**0.77**

**FIGURE 14:**
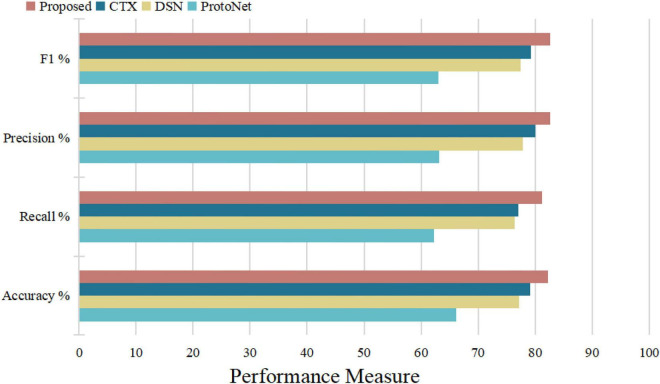
Performance comparison with various models.

Figure 14 illustrates the algorithm’s performance based on the above test parameters. The proposed FRN can be predicted to be the best due to its property of classifying affected regions spread over a given image from a performance overview. 3D ResNet guarantees its performance in computation time and average accuracy for medical image datasets, with the highest recall and satisfying precision. Statistical, visual, and experimental evidence is provided through comparisons with other algorithms.

To sum up, through the above experiments, we can see that the performance of the ProtoNet method is not as good as other methods. Because traditional ProtoNet algorithms extract feature histograms through direct statistical methods, which are linear features that need to be combined with non-linear classifiers to perform well. The DSN method outperforms the ProtoNet method, probably because the DSN algorithm predicts class membership by computing the distances between query points and their projections into the latent subspace formed by the supporting images of each class, which improves methods for image predictive classification. Whereas the CTX method explicitly produces class-level linear reconstructions and outperforms the DSN method. Our algorithm decomposes the image into multi-scale layers and performs 3D residual network feature extraction and feature map reconstruction to predict classification, greatly enhancing the discrimination of image feature representation. Therefore, our algorithm has the best average classification accuracy, specificity, and sensitivity, which indicates that 3D FRN-ResNet indeed improves image classification accuracy. On the one hand, our algorithm proposes a 3D residual connection network, which extends the idea of residual connections to three dimensions. It makes full use of the spatial and contextual information of the image and preserves the spatial details when converting the extracted features into vectors and location information. Thus, higher average accuracy than other methods is achieved, demonstrating the effectiveness of the 3D residual connection network and classification based on feature map reconstruction. On the other hand, since we decompose the image into multiple-scale layers, sufficient scale information is used when generating multi-scale visual histograms. Therefore, our algorithm has the best classification specificity and sensitivity.

## Discussion

The 3D FRN-ResNet proposed in this paper can effectively improve the identification accuracy and recall rate of sMRI data from MDD patients and healthy controls, and verifies its effectiveness and feasibility. The proposed model can assist physicians to complete the diagnosis, and has significant significance in research value.

The method is compared with some typical medical image classification algorithms, and the results are shown in Table 4. All of these methods use the private SHH dataset. These results can be compared with those obtained using the proposed method. Our proposed method is one of the best and achieves better performance than other methods evaluated under the same conditions. Jiao et al. (2017) introduced a joint model with a CNN layer and a parasitic metric layer. Where the CNN layer provides the essential discriminative representation, and the metric learning layer enhances the classification performance for that particular task (Jiao et al., 2017). An et al. (2021) proposed a multi-scale convolutional neural network, a medical classification algorithm based on a visual attention mechanism, which automatically extracts high-level discriminative appearance features from the original image. In the method proposed by Ben et al. (2020), a new classification framework was developed to classify medical images using sparse coding and wavelet analysis, which showed a significant improvement in identification accuracy. Cheng et al. (2022) proposed a modular group attention block that captures feature dependencies in medical images in both channel and spatial dimensions for resulting in improved classification accuracy. Abdar et al. (2021) proposed a novel, simple and effective fusion model with uncertainty-aware module for medical image classification called Binary Residual Feature fusion (BARF).

**TABLE 4:** Results comparison with typical methods.

Method	Accuracy	Recall	Precision	*F*1
Jiao et al., 2017	0.82	0.79	**0.84**	0.81
An et al., 2021	0.81	0.79	0.82	0.80
Ben et al., 2020	0.79	0.77	0.81	0.79
Cheng et al., 2022	0.83	0.80	0.82	0.81
Abdar et al., 2021	0.81	0.80	0.81	0.79
**Proposed**	**0.85**	**0.82**	0.82	**0.82**

*Bold values mean the best performance.*

Table 4 shows that the model has some advantages in classification. The bold text in the table represents the best performance. But there are still differences in accuracy, and the model has limitations. In future work, solutions can be proposed for this situation, such as designing a network structure more suitable for small samples to maximize the neural network learning ability. In addition, many of the algorithms proposed in the top methods have excellent performance. How to combine the advantages of these algorithms and integrating them into models is the focus of future work. In clinical care, it helps experts understand patients’ current situation faster and more accurately, saving experts’ time and achieving a leap in the quality of automatic medical classification.

## Conclusion

This paper proposes an automated MDD sMRI data identification framework and performs a performance validation on the private SHH dataset with satisfactory results. The framework comprises a feature extractor and a feature map reconstruction network. 3D-ResNet acts as a feature extractor to ensure that MDD sMRI data with depth features can be learned. Then, the feature map reconstruction network solving the reconstruction problem in a closed-form produces a class of simple and powerful characters, which contains fine spatial details without overfitting the position or pose. Furthermore, we use an auxiliary loss that encourages support features from different classes to span the potential space to more clearly distinguish between classes. Additionally, a benchmarking clinical MDD sMRI images dataset with 68 subjects (SHH) is collected to train and test the model, and we evaluate the proposed 3D FRN-ResNet on the SHH dataset. Experimental results show that the proposed model exhibits promising performance and outperforms the typical other methods, achieving the accuracy, recall, precision, and *F*1 values of 0.86776, 0.84237, 0.85333, and 0.84781, respectively. Compared with some benchmark methods, the method proposed in this paper can effectively improve the identification accuracy and recall of MDD and healthy controls, and then assist doctors to complete the diagnosis in medicine, which has great value in practical clinical computer-aided diagnosis applications.

Even though the 3D FRN-ResNet framework has demonstrated its potential within the automated identification for MDD sMRI data, some limitations still need to be improved. For example, the model performance cannot be well exploited due to sample size limitations. Thus, we can use better data enhancement methods, which provide a good starting point for further research.

## Data Availability Statement

The datasets presented in this article are not readily available because the raw/processed data required to reproduce these findings cannot be shared at this time as the data also forms part of an ongoing study. Requests to access the datasets should be directed to XL, dmia_lab@zcmu.edu.cn.

## Author Contributions

JH, YH, and XL conceived and designed the study. JH, JY, JW, XX, and XL contributed to the literature search. JH, YH, YW, and YL contributed to data analysis and data curation. JH, JY, JZ, RL, and XL contributed to data visualization. JH and JK contributed to software implementation. JH, YH, JY, JW, XX, and XL contributed to the tables and figures. JH, YW, YL, RL, JK, and XL contributed to the writing of the report. JH, JY, YW, RL, and XL contributed to review and editing. All authors have read and approved the publication of this work.

## Conflict of Interest

The authors declare that the research was conducted in the absence of any commercial or financial relationships that could be construed as a potential conflict of interest.

## Publisher’s Note

All claims expressed in this article are solely those of the authors and do not necessarily represent those of their affiliated organizations, or those of the publisher, the editors and the reviewers. Any product that may be evaluated in this article, or claim that may be made by its manufacturer, is not guaranteed or endorsed by the publisher.
